# An International Survey of Peritoneal Dialysis Exercise Practices and Perceptions

**DOI:** 10.1016/j.ekir.2023.04.024

**Published:** 2023-05-03

**Authors:** Paul N. Bennett, Clara Bohm, Angela Yee-Moon Wang, Talerngsak Kanjanabuch, Ana Elizabeth Figueiredo, Oksana Harasemiw, Leanne Brown, Iwona Gabrys, Dev Jegatheesan, Kelly Lambert, Courtney J. Lightfoot, Jennifer MacRae, Nicole Scholes-Robertson, Krista Stewart, Brett Tarca, Nancy Verdin, Madeleine Warren, Mike West, Deborah Zimmerman, Jeannette Finderup, Emilie Ford, Heitor S. Ribeiro, Qunyan Xu, Stephanie Thompson

**Affiliations:** 1 School of Nursing and Midwifery, Menzies Health Institute Queensland, Griffith University, Queensland, Australia; 2 Satellite Healthcare, USA; 3 Chronic Disease Innovation Center, Manitoba, Canada; 4 Medicine/Nephrology, University of Manitoba, Manitoba, Canada; 5 Queen Mary Hospital, The University of Hong Kong, Hong Kong SAR, China; 6 Division of Nephrology and Center of Excellence in Kidney Metabolic Disorders, Faculty of Medicine, Chulalongkorn University, Thailand; 7 Graduate Program in Medicine and Health Sciences, Pontifícia Universidade Católica do Rio Grande do Sul Escola de Ciências da Saúde e da Vida, Brazil; 8 Queensland University of Technology, Queensland, Australia; 9 Alberta Kidney Care North, Alberta Health Services, Alberta, Canada; 10 The University of Queensland, Queensland, Australia; 11 Princess Alexandra Hospital, Brisbane, Queensland, Australia; 12 University of Wollongong, Wollongong, New South Wales, Australia; 13 Department of Health Sciences, College of Medicine, Biological Sciences and Psychology, University of Leicester, UK; 14 University of Calgary, Alberta, Canada; 15 University of Sydney, Sydney, New South Wales, Australia; 16 Manitoba Renal Program, Manitoba, Canada; 17 Alliance for Research in Exercise, Nutrition and Activity, University of South Australia, South Australia, Australia; 18 The Global Renal Exercise Network Patient Engagement Council, Canada; 19 Warren-Charnock Associates, UK; 20 University of California Davis, California, USA; 21 Department of Medicine, Ottawa Hospital, Ontario, Canada; 22 Aarhus University Hospital, Denmark; 23 Research Center in Sports Sciences, Health Sciences and Human Development, University of Maia, Portugal; 24 University Center ICESP, Brazil; 25 Clinical and Health Sciences, University of South Australia, South Australia, Australia; 26 Division of Nephrology, University of Alberta, Alberta, Canada

**Keywords:** exercise, exit site, kidney failure, nephrologist, peritoneal dialysis, physical activity

## Abstract

**Introduction:**

Low activity levels and poor physical function are associated with technique failure and mortality in people receiving peritoneal dialysis (PD). Adequate levels of physical function are required to maintain independence for people choosing this predominantly home-based therapy. The objective of this study was to identify the exercise-related perceptions and practices of PD clinicians globally.

**Methods:**

We conducted a cross-sectional survey of PD clinicians from English-, Thai-, Spanish-, and Portuguese-speaking PD-prevalent countries exploring clinicians’ perceptions and practices of swimming, activity following PD catheter insertion, lifting, and falls prevention. This study was convened by the International Society of Peritoneal Dialysis and Global Renal Exercise Network between July and December 2021.

**Results:**

Of 100 of the highest PD-prevalent countries, 85 responded and were represented in the findings. A total of 1125 PD clinicians (448 nephrologists, 558 nephrology nurses, 59 dietitians, and 56 others) responded from 61% high-income, 32% upper middle-income and 7% lower middle-income countries. The majority (*n* = 1054, 94%) agreed that structured exercise programs would be beneficial for people receiving PD. Most respondents believed people on PD could perform more exercise (*n* = 907, 81%) and that abdominal strengthening exercises could be safely performed (*n* = 661, 59%). Compared to clinicians in high-income countries, clinicians from lower middle-income status (odds ratio [OR], 5.57; 1.64 to 18.9) are more likely to promote participation in physical activity.

**Conclusion:**

Clinicians know the importance of physical activity in people receiving PD. Exercise counseling and structured exercise plans could be included in the standard care of people receiving PD to maintain independence.

## Graphical abstract

**Figure:**
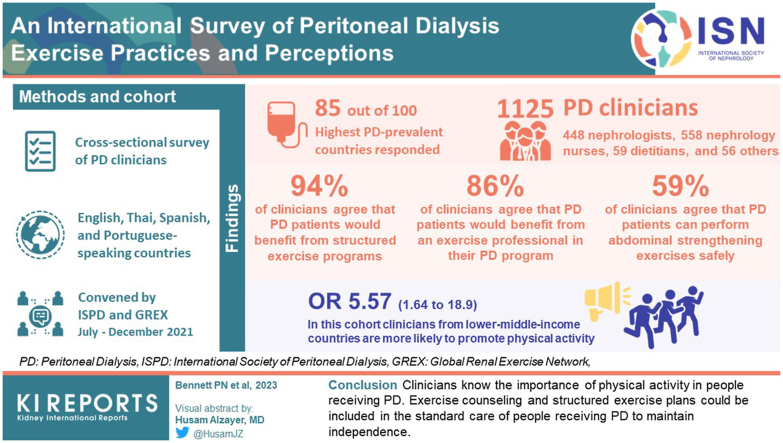

Low physical activity levels and functional impairment are highly prevalent in people receiving PD.^1^^,^^2^ Functional impairment is associated with PD technique failure and mortality.^3^^,^^4^ Frailty and falls are common in the PD population and are associated with increased mortality.^5^

Life participation, which incorporates physical activity and physical function, is a key patient-important outcome for people receiving PD.^6^ The potential benefits of exercise and physical activity for people receiving PD is shown in Table 1. Although these benefits are possible, the evidence to support the benefits has not achieved great rigor and still requires better evidence to assert major improvements in physical function and mental health.^7^ In saying that, many people receiving PD are keen to participate in physical activity and exercise programs.^8^ However, symptoms of kidney disease and the permanent presence of the PD catheter can affect participation levels and intensity of exercise.^9^^,^^10^

**Table 1:** Benefits of exercise and physical activity in people receiving peritoneal dialysis

Musculoskeletal
Improve and maintain muscle strength and muscle mass
Reduce intramuscular fat
Enhance fiber II motor neuron activation
Reduce falls risk
Cardiovascular
Improve and maintain cardiorespiratory fitness
Increase exercise tolerance
Stabilize blood pressure
Reduce left ventricular mass
Minimize cardiometabolic diseases risk
Metabolic
Improve weight management
Enhance CKD-mineral bone axis parameters
Improve insulin sensitivity and lipid profile
Symptom Burden
Improve sleep quality
Reduce constipation
Reduce fatigue
Reduce restless legs syndrome
Psychosocial wellbeing
Improve and maintain ability to perform activities of daily living
Increase activation and engagement in self-management behaviors
Enhance cognition
Enhance mood

CKD, chronic kidney disease.

Given that most PD clinicians are not trained exercise professionals, guidance is required from multidisciplinary sources regarding physical activity and exercise advice for the PD population.^11^ However, access to and engagement of exercise professionals are not a part of regular clinical treatment in the majority of PD programs.^2^ This is in the context of the well-known benefits of physical activity in people receiving PD (Table 1).

People receiving PD are required to possess a level of physical function to carry dialysis fluid, set up PD manually or with cyclers and empty drain bags.^12^ However, there is a lack of evidence about the role PD clinicians play in the promotion of physical activity behaviors of individuals receiving PD. Furthermore, little is known about the variability in global practices related to exercise and physical activity promotion in the PD population.^13^ Although country-based and regional studies have provided some insights into exercise practices and perceptions in PD,^9^^,^^14^ a broader global understanding has not been reported. Therefore, this survey aimed to identify the exercise perceptions and practices of PD clinicians around the world to inform future practice and research.

## Methods

### Survey Administration

This was a cross-sectional web-based survey. To be able to participate and meet the inclusion criteria, respondents were required to state that they were providing clinical care to people receiving PD. We used convenience sampling to capture the perspectives of health professionals from 100 PD-prevalent countries.^15^^,^^16^ This consisted of emailing professional national nephrology societies that were affiliated and known to the International Society of Peritoneal Dialysis and the International Society of Nephrology to assist the facilitation and dissemination of the survey to the relevant sample. This study was reported according to the EQUATOR Consensus-Based Checklist for Reporting of Survey Studies (Supplementary Table S2).^17^

### Data Collection

The survey consisted of a bespoke 13-item online questionnaire that was developed through expert consensus with 20 international PD clinicians, exercise professionals, and people receiving PD (Supplementary Table S1). A pilot questionnaire was sent to 10 global PD clinicians whose feedback resulted in minor survey revisions, such as adding comment boxes to most questions. The final survey had 13 questions grouped into 3 sections. Section 1 (Questions 1 and 2) asked for details about the PD program service related to exercises for people receiving PD. Section 2 had 9 questions, which explored clinicians’ practice (Question 3) and perceptions on exercise in people receiving PD (Question 4–Question 11). Specifically, Question 3 asked clinicians’ practice on advising different types of exercises such as swimming, postcatheter insertion activity, lifting, and falls prevention. Responses including “yes,” “no,” “don’t know” were provided. Question 4 to Question 11 asked clinicians’ perceptions on exercise in people receiving PD. A 5-point Likert scale was provided with 1 indicating “strongly agree,” and 5 “strongly disagree.” Section 3 included 2 open-ended questions soliciting clinicians’ perceptions otherwise not captured. The survey used a 5-point Likert scale with additional open-ended questions following each question. The survey was originally developed in English and then subsequently translated into Thai, Portuguese, and Spanish using the back-translation method. The project was approved by the University of South Australia Human Research Ethics Committee (ID204071). All individual participants were offered an information page at the beginning of the web-based survey. Completing the survey indicated consent.

This questionnaire was disseminated through International Society of Peritoneal Dialysis, International Society of Nephrology, as well as regional and national professional nephrology societies. We administered the survey online via SurveyMonkey (www.surveymonkey.com) from July 2021 to December 2021. A standard message with the information of the study and the weblink to the survey was provided through the professional networks: “The aim of this survey is to explore global PD exercise and physical activity to inform PD exercise recommendations. By completing this survey, you are consenting to your de-identified information being analyzed. If you wish to receive a copy of the results, please insert your email address at the end of this survey.”

### Data Analysis

Participants’ practice on advising various types of physical activity in the current cohort was described using frequency and proportion. In describing participants’ perceptions about exercise, the 5-point Likert scale was first grouped into 3 categories, which were “Strongly agree/ Agree,” “Neutral,” and “Strongly disagree/disagree.” The proportion of each category was then reported.

The analysis of the association between clinicians’ characteristics (exposure variables) and practice and perceptions (outcome variables) toward exercise among patients on PD was completed by fitting a binomial generalized estimation equation. Before fitting the binomial generalized estimation equation, clinicians’ practice and perceptions were both dichotomized, specifically “yes” and “otherwise” for practice outcomes, and “agree” and “otherwise” for perception outcomes. An exchangeable correlation structure was chosen to model the intracountry correlation in clinicians’ practice and perception. Interaction terms were not investigated in this study for the lack of *priori* subject-knowledge in this respect. Regarding occupation, nephrologists were set as the reference group that allowed a contrast in perceptions between physician and nursing or allied health professionals. Complete case analysis was performed after excluding the 2 incomplete responses. Analysis was performed in R Statistical Software (v4.1.2; R Core Team 2021). Free text responses were categorized to assist in explaining the responses and providing further depth to the quantitative responses.

## Results

On a nationwide-based response rate, 85 of 100 PD-prevalent countries contacted responded to the survey. We received complete responses from 1125 PD clinicians with most respondents from Asia 35% (*n* = 388), followed by Europe 24% (*n* = 265), North America 19% (*n* = 211), Australia/New Zealand 13% (*n* = 149), Latin America 7% (*n* = 83), Middle East 2% (*n* = 24), and Africa <1% (*n* = 5) (Table 2).

**Table 2:** Participant characteristics representing global peritoneal clinician responses

Characteristic	Frequency	Percentage
Continent		
Asia	388	34.49
Europe	265	23.56
North America	211	18.76
Australia/New Zealand	149	13.24
Latin America	83	7.38
Middle East	24	2.13
Africa	5	0.44
Country Income status		
High-income	682	60.62
Upper middle-income	362	32.18
Lower middle-income	81	7.20
Low-income	0	0
Gender		
Female	810	72.0
Male	315	28.0
Profession		
Nurse	558	49.60
Nephrologist	448	39.82
Dietitian	59	5.24
Exercise professional	28	2.49
Social worker	14	1.24
Other	18	1.60
Work experience		
Less than 2 yr	103	9.16
2–5 yr	203	18.04
More than 5 yr	819	72.80
Program patient numbers		
Less than 20	195	17.33
20–50	314	27.91
51–100	258	22.93
More than 100	358	31.82

Nurses (*n* = 558, 50%), nephrologists (*n* = 448, 40%), dietitians (*n* = 59, 5%) and exercise professionals (*n* = 28, 3%) made up the largest response groups. Others included social workers, patient care technicians, and pharmacists. 72% of respondents were female. Most respondents were from high-income countries (61%), followed by 32% from upper middle-income countries, 7% from lower middle-income countries, with no respondents from low-income countries. The majority had greater than 10 years’ clinical experience (73%) with only 9% less than 2 years’ experience. Similar numbers of participants worked in programs with fewer than (45%) and greater than 50 patients (55%) (Table 2).

**Figure 1:**
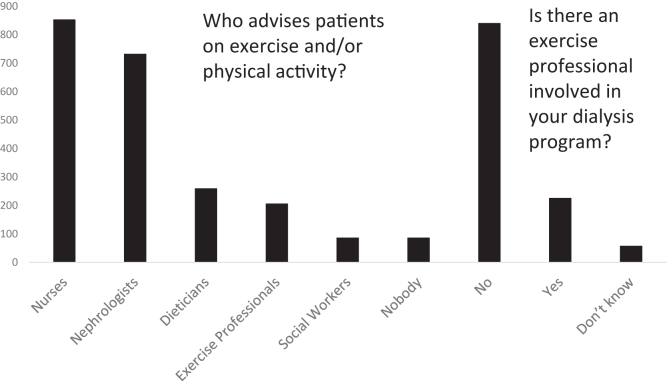
Clinicians who advise the patients on exercise and physical activity and the number of exercise professionals involved in dialysis programs (*N* = 1125).

Nurses (80%), nephrologists (65%), dietitians (23%) and exercise professionals (18%) were the main providers of exercise advice to patients (respondents were able to choose more than 1 option). In 8% of clinics no one provided exercise advice to patients and 20% of participants’ clinics had access to exercise professionals (Figure 1).

Most clinicians provided recommendations for lifting (59%), exercise postcatheter insertion (73%), and falls prevention (66%), with 50% providing recommendations on swimming. Of the 664 respondents stating that they provide recommendations for lifting, 119 reported lifting restrictions ranging from avoiding lifting of loads of more than 13 kg (25 lbs) to advice of no more than 26 kg (50 lbs). From the 822 respondents stating they provide postcatheter insertion recommendations, the most common advice provided was to avoid or limit physical activity and/or lifting within the first 6 weeks post PD catheter insertion. From the 745 reporting falls prevention advice, 40 qualitative responses indicated this was mostly in the form of specifically offered training in falls prevention training (98% of responses) and walking aid training (10% of responses). Half of all respondents (556) reported offering swimming advice. In further comments, 60 respondents reported recommending patients avoid watersports and swimming with 62 responses reporting that swimming should only be done in a private pool or ocean. Those offering swimming advice recommended that swimming should be performed with a colostomy bag or other form of waterproof dressing, and that postcatheter exit site care was required.

Walking was the most frequently reported aerobic activity recommended by clinicians followed by cycling, jogging, formal aerobic classes, stretching, swimming, resistance training, dance, Tai Chi, and Chinese boxing. Free text responses included some support for vigorous exercise to be performed after the abdomen has been emptied of peritoneal dialysate.

**Figure 2:**
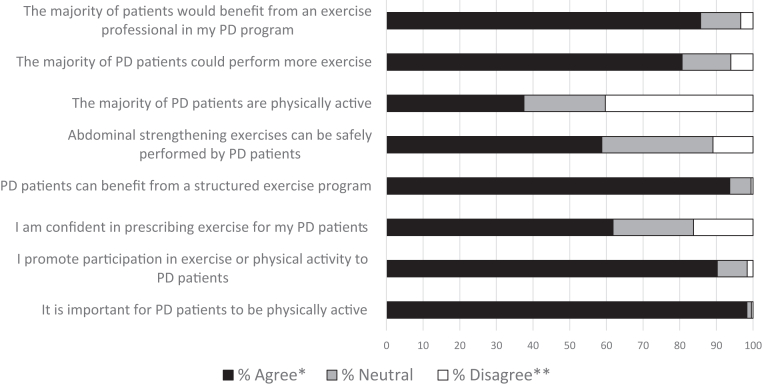
Clinicians’ perceptions and practices toward exercises in peritoneal dialysis patients showing overwhelmingly positive perceptions toward exercise and physical activity (*N* = 1125). ∗Strongly Agree and Agree combined: ∗∗Strongly Disagree and Disagree combined. PD, peritoneal dialysis.

The overwhelming majority agreed that it is important for people receiving PD to be physically active (strongly agreed/agreed = 98%), that they promote exercise or physical activity to their patients (90%), that people receiving PD can benefit from a structured exercise program (94%), that most patients could perform more exercise (81%), and that most would benefit from an exercise professional in their PD program (86%). Responses varied from the perceptions of how physically active patients on PD are, with 38% agreeing that most patients on PD are physically active, 40% disagreeing, and 22% neutral or not sure. Most respondents agreed that abdominal strengthening exercises can be safely performed by patients on PD (59%) and that they were confident in prescribing exercise (62%) (Figure 2).

Compared to nephrologists and across countries, dieticians had lower odds of being confident in prescribing exercise (OR, 0.33; 95% confidence interval [CI] 0.19–0.55) on average. Compared to nephrologists, dietitians perceived that fewer people on PD were physically active (OR, 0.34; 95% CI 0.13–0.90). Clinicians with more than 5 years’ clinical experience were more confident in prescribing exercises compared to those with less than 2 years’ experience (OR, 2.04; 95% CI, 1.30, 3.20) averaging across the surveyed countries.

Averaged across countries, gender was associated with clinicians’ perception when asked “I promote participation in exercise or physical activity to peritoneal dialysis patients.” Male clinicians were less likely to promote participation in exercise or physical activity compared to female clinicians after adjusting for occupation (OR, 0.43; 95% CI, 0.26–0.73). They were also less likely to think that patients on PD would benefit from exercise professional involvement (OR, 0.60; 95% CI, 0.39–0.94).

Country income status was associated with clinicians’ perceptions of PD physical activity. Compared to clinicians in high-income countries, clinicians from lower middle-income countries (OR, 5.57; 1.64 to 18.9) were more likely to promote participation in exercise and physical activity (Table 3). There were variations in the provision of recommendations for lifting, swimming, activity following catheter insertion and falls prevention. Evidence suggested that programs from higher income countries with greater resources were likely to provide more exercise recommendations (Table 4). The complete table of distribution of exercise perception outcomes by covariates can be found in Supplementary Table S3 (Supplementary Data).

**Table 3:** Association of occupation, gender, years worked, program size, and country income status with clinicians’ perceptions toward exercise and physical activity in patients on peritoneal dialysis

Participant characteristics	It is important for PD patients to be physically active OR (95% CI)	*P*-value	I promote participation in exercise or physical activity to PD patients OR (95% CI)	*P*-value	I am confident in prescribing exercise for my PD patients OR (95% CI)	*P*-value	PD patients can benefit from a structured exercise program OR (95% CI)	*P*-value
Gender		0.643		0.002		0.989		0.830
Female	Ref		Ref		Ref		Ref	
Male	0.91 (0.66, 1.25)		0.43 (0.26, 0.73)		1.00 (0.68, 1.47)		1.07 (0.58, 1.99)	
Occupation		n/a		n/a		<0.001		n/a
Nephrologist	Ref		Ref		Ref		Ref	
Dietician	--		0.91 (0.50, 1.67)		0.33 (0.19, 0.55)		0.94 (0.52, 1.69)	
Exercise professional	--		1.62 (0.35, 7.40)		9.96 (1.96, 50.5)		--	
Nurse	0.94 (0.74, 1.20)		0.70 (0.46, 1.08)		1.14 (0.76, 1.70)		1.08 (0.51, 2.27)	
Social worker	--		--		0.46 (0.23, 0.90)		--	
Other	--		0.28 (0.11, 0.73)		0.93 (0.42, 2.06)		--	
Work experience		0.039		0.301		0.006		0.322
<2 yr	Ref		Ref		Ref		Ref	
2–5 yr	1.37 (0.98, 1.92)		1.44 (0.87, 2.39)		1.37 (0.93, 2.03)		2.00 (0.79, 5.06)	
>5 yr	1.45 (1.00, 2.10)		1.68 (0.85, 3.33)		2.04 (1.30, 3.20)		1.77 (0.81, 3.87)	
Size of center		0.885		<0.001		<0.001		0.017
<20 patients	Ref		Ref		Ref		Ref	
20–50 patients	1.17 (0.71,1.93)		2.54 (1.42, 4.55)		1.95 (1.50, 2.53)		1.03 (0.52, 2.03)	
51–100 patients	0.95 (0.63,1.43)		1.46 (0.93, 2.29)		1.51 (1.05, 2.18)		2.18 (0.93, 5.12)	
>100 patients	0.99 (0.68, 1.42)		2.44 (1.57, 3.78)		1.56 (1.06, 2.30)		2.03 (1.04, 3.96)	
Income status		n/a		0.024		0.003		0.119
High-income	Ref		Ref		Ref		Ref	
Upper middle-income	0.71 (0.52, 0.96)		1.20 (0.50, 2.89)		2.14 (1.34, 3.41)		1.58 (0.75, 3.33)	
Lower middle-income	--		5.57 (1.64, 18.9)		1.95 (0.81, 4.79)		4.01 (0.93, 17.2)	

Participant characteristics	Abdominal strengthening exercises can be safely performed by peritoneal dialysis patients OR (95% CI)	*P*-value	The majority of peritoneal dialysis patients are physically active OR (95% CI)	*P*-value	The majority of peritoneal dialysis patients could perform more exercise OR (95% CI)	*P*-value	The majority of patients would benefit from an exercise professional in my peritoneal dialysis program OR (95% CI)	*P*-value
Gender		0.473		0.847		0.718		0.027
Female	Ref		Ref		Ref		Ref	
Male	1.12 (0.82, 1.53)		1.02 (0.80, 1.31)		1.07 (0.74, 1.55)		0.60 (0.39, 0.94)	
Occupation		<0.001		0.021		0.002		0.188
Nephrologist	Ref		Ref		Ref		Ref	
Dietician	0.32 (0.18, 0.56)		0.34 (0.13, 0.90)		0.67 (0.42, 1.08)		1.07 (0.45, 2.57)	
Exercise professional	1.44 (0.69, 3.02)		0.96 (0.38, 2.42)		2.51 (0.49, 13.0)		1.29 (0.47, 3.59)	
Nurse	0.57 (0.40, 0.83)		0.88 (0.60, 1.30)		0.70 (0.49, 1.01)		0.77 (0.49, 1.20)	
Social worker	0.32 (0.13, 0.79)		1.60 (1.11, 2.31)		1.32 (0.73, 2.38)		1.68 (0.87, 3.23)	
Other	0.50 (0.20, 1.27)		0.59 (0.21, 1.64)		0.83 (0.28, 2.47)		1.06 (0.26, 4.30)	
Work experience		0.742		0.162		0.828		0.102
<2 years	Ref		Ref		Ref		Ref	
2 to 5 years	1.24 (0.71, 2.15)		0.78 (0.40, 1.53)		0.87 (0.52, 1.45)		1.37 (0.90, 2.08)	
>5 years	1.19 (0.73, 1.96)		1.01 (0.50, 2.03)		0.94 (0.54, 1.62)		0.96 (0.53, 1.71)	
Size of center		0.609		0.908		0.079		0.861
<20 patients	Ref		Ref		Ref		Ref	
20−50 patients	1.10 (0.76, 1.60)		0.88 (0.60, 1.29)		1.62 (1.04, 2.52)		0.89 (0.57, 1.38)	
51−100 patients	1.12 (0.74, 1.71)		0.90 (0.61, 1.33)		1.65 (1.06, 2.58)		1.09 (0.67, 1.79)	
>100 patients	1.22 (0.90, 1.65)		0.87 (0.58, 1.31)		1.81 (1.04, 3.15)		0.91 (0.52, 1.60)	
Income status		0.036		0.162		0.313		0.05
High-income	Ref		Ref		Ref		Ref	
Upper middle-income	2.07 (1.19, 3.59)		0.80 (0.39, 1.63)		0.82 (0.42, 1.59)		1.08 (0.65, 1.80)	
Lower middle-income	1.12 (0.63, 1.98)		2.12 (0.85, 5.27)		0.59 (0.30, 1.16)		2.22 (1.15, 4.28)	

CI, confidence interval; OR, odds ratio; Ref, x.

**Table 4:** Association of occupation, gender, years worked, program size, and country income status with clinicians’ recommendations on lifting, swimming, postcatheterization, and falls prevention

Participant characteristics	Lifting OR (95% CI)	*P*-value	Swimming/water sports OR (95% CI)	*P*-value	Activity after catheter insertion OR (95% CI)	*P*-value	Fall prevention OR (95% CI)	*P*-value
Gender		0.532		0.119		0.363		0.944
Female	Ref		Ref		Ref		Ref	
Male	0.88 (0.60, 1.30)		0.79 (0.59, 1.06)		1.19 (0.82,1.71)		0.99 (0.75,1.30)	
Occupation		<0.001		<0.001		<0.001		<0.001
Nephrologist	Ref		Ref		Ref		Ref	
Dietician	0.25 (0.07, 0.91)		0.15 (0.06, 0.36)		0.15 (0.07,0.34)		0.64 (0.37,1.12)	
Exercise professional	1.27 (0.40,4.04)		0.18 (0.05, 0.67)		0.92 (0.38,2.18)		1.41 (0.51,3.86)	
Nurse	1.45 (0.91,2.28)		0.98 (0.65, 1.49)		1.27 (0.99,1.63)		3.38 (2.31,4.93)	
Social worker	1.85 (1.26, 2.73)		0.66 (0.26, 1.72)		1.04 (0.43,2.50)		2.10 (1.20,3.67)	
Other	0.07 (0.01, 0.55)		0.06 (0.01, 0.47)		0.19 (0.06,0.64)		1.76 (0.53,5.83)	
Work experience		0.377		<0.001		<0.001		0.540
<2 yr	Ref		Ref		Ref		Ref	
2–5 yr	1.30 (0.72,2.35)		1.42 (0.78, 2.57)		1.67 (1.13,2.48)		0.82 (0.53,1.26)	
>5 yr	1.54 (0.80,2.98)		1.94 (1.17, 3.21)		2.47 (1.62,3.79)		0.94 (0.56,1.57)	
Size of center		0.293		<0.001		0.002		0.172
<20 patients	Ref		Ref		Ref		Ref	
20−50 patients	1.16 (0.72,1.87)		1.65 (1.20, 2.27)		1.44 (0.98,2.11)		1.27 (0.85,1.90)	
51−100 patients	1.48 (0.87,2.54)		1.43 (0.93, 2.19)		1.97 (1.38,2.80)		1.11 (0.75,1.65)	
>100 patients	1.41 (0.88,2.26)		1.02 (0.73, 1.43)		1.76 (1.24,2.49)		1.63 (1.06,2.53)	
Income status		<0.001		<0.001		<0.001		<0.001
High-income	Ref		Ref		Ref		Ref	
Upper middle-income	0.16 (0.07,0.37)		0.24 (0.12,0.50)		0.31 (0.17,0.56)		2.04 (1.26,3.31)	
Lower middle-income	0.58 (0.28,1.18)		0.19 (0.08,0.46)		0.64 (0.27,1.50)		2.93 (1.54,5.55)	

CI, confidence interval;OR, odds ratio; Ref, reference.

## Discussion

This study is the first to explore international practices and perceptions related to physical activity and exercise in PD programs. We found overwhelming consensus regarding the importance of physical activity for people on PD. However, this interest in physical activity contrasts with the many studies showing low activity^18^, ^19^, ^20^, ^21^ and functional impairment in people on PD.^16^^,^^18^^,^^22^^,^^23^ This disconnect between clinician interest and low physical activity and function in people receiving PD requires exploring. This is likely related to the behavioral change that is required to increase physical activity,^24^ which may require more than what PD clinicians can offer because they may lack expertise in providing individualized, prescriptive exercise programs. This expertise is particularly important given the challenges when prescribing exercise for people who are required to live with a permanent peritoneal catheter and endure the challenging side effects of kidney disease.^25^ To overcome this, exercise professionals (e.g., exercise physiologists, kinesiologists, physical therapists) who are trained in exercise and behavioral change could be added as members of the kidney care team.

We found regional differences between clinicians’ perceptions of the activity levels of people on PD. Respondents from Thailand and Malaysia perceived greater activity levels in their patient cohorts. This may be due to the younger mean age of people receiving PD in these countries^26^ and thus higher levels of physical activity. However, it is also feasible that there is a culture where clinicians promote exercise to develop more independence following the commencement of PD.^27^^,^^28^

Our study highlighted that income status of countries had an association with exercise and physical activity perceptions and practices. Those in lower income countries were less likely to advise on lifting, swimming, exercise postcatheter insertion, and fall prevention. It is likely that in less resourced communities, water quality and public swimming center hygiene standards may necessitate this reluctance for swimming to be encouraged. Therefore, swimming activity practice recommendations require regional context.^29^^,^^30^

An unexpected finding from our study identified dietitians as less likely than nephrologists and nurses to perceive that people receiving PD are active. Dietitians’ perceptions align more closely with studies quantifying the low physical activity and function of people receiving PD.^7^ It is likely that dietitians have a more informed understanding of the activity status of a person receiving PD given that they measure metrics such as energy balance during assessments and can quantify how inactive people are. In addition, dietitians perceived that they are less confident in prescribing exercise than nephrologists, nurses, and exercise professionals. Dietitians are trained to encourage exercise, but the ‘prescribing’ part is not in dietitians’ scope of practice other than general recommendations. In many countries, dietitians are not available^31^ and are unable to refer to an exercise professional or prescribe exercise interventions.^32^

We found that female clinicians promoted exercise more than their male colleagues. This aligns with studies reporting that female physicians are more patient-centric and more likely to promote preventive care compared to their male counterparts.^33^, ^34^, ^35^ Importantly, we had a greater response from females in the survey, reflecting both a large proportion of female nursing, dietitian, and social worker respondents, but also reflecting the larger female nephrologist ratio response.

This study has several key clinical implications. First, we provide additional evidence to support the perception that PD clinicians provide exercise advice that contrasts with low activity levels^7^ and poor physical function.^18^ Second, there may be a need to create formal training for those health professionals wishing to provide dialysis exercise therapy. Third, country wealth and income status need to be considered when developing physical activity and exercise policies. Finally, strategies to target and engage male clinicians to prioritize exercise and physical activity are required.

Our study had specific strengths and limitations. The strengths of this study included its simplicity and brevity (13 questions) and the inclusion of a diverse international sample including non-English speaking respondents. The perceptions and practices of clinicians from non-English speaking countries have not been represented in global PD exercise surveys previously. However, the program or center where the respondent worked was not identifiable to obtain a more in-depth understanding of responses. The survey was limited by the convenience sampling method using international and national nephrology societies to disseminate the survey to their members. The lack of sampling weight becomes an obstacle for the generalizability of the results. Survey participation was voluntary and likely to represent those with an interest in physical activity and exercise that may lead to selection bias. There were fewer responses from low-income countries, which is likely related to a lower number of PD programs in these regions. Finally, to encourage honest and accurate responses and minimize response bias, we chose not to collect data that would identify individuals, individual centers, or workplaces. This limited our capacity to analyze data at a center level and the number of respondents from the same center.

In conclusion, this study demonstrates consensus among PD clinicians regarding the importance of physical activity and exercise in people receiving PD. Encouragement and motivation from PD clinicians is applauded and encouraged; however, many people still have low physical function and low activity levels. We believe that PD programs consider physical function and independence as outcomes of importance. We recommend that strategies such as nephrology practitioner exercise education, specific exercise counseling, and the inclusion of exercise professionals in standard care may be required to increase physical activity, improve physical function, and maintain independence.

## Disclosure

PNB has received honoraria from VIFOR, GSK, and is a consultant to Satellite Healthcare. AEF has received honoraria from Baxter and Bayer. LB has received honoraria from Astra Zeneca, Amgen, and Otsuka. TK reports grants from Thailand Science Research and Innovation Fund Chulalongkorn University, Thailand, during the conduct of the study; personal fees from VISTERRA and ELEDON; and personal fees from AstraZeneca and Baxter Healthcare outside the submitted work. CB has received research funding from Hope Pharmaceuticals and has ownership interest in Precision Advanced Digital Manufacturing Inc. DZ has received honoraria from Bayer and Otsuka. KL reports honoraria from Otsuka and consultant to Abbott Nutrition International. All other authors have declared no competing interests.

## Supplementary Material

Supplementary File (PDF)

**Table S1.**Survey (English): The exercise perceptions and practices of peritoneal dialysis clinicians.

**Table S2.** EQUATOR Consensus-Based Checklist for Reporting of Survey Studies.

**Table S3.** Distribution of exercise perception outcomes by covariates.
