# Supplementary material for: An International Survey of Peritoneal Dialysis Exercise Practices and Perceptions
Source: Kidney Int Rep. 2023 May 3;8(7):1389–98. doi: 10.1016/j.ekir.2023.04.024 (PMC10334400; doi:10.1016/j.ekir.2023.04.024)
Supplement: Supplementary File (PDF) [file mmc1.pdf]

Supplementary Table 1. Survey (English): The exercise perceptions and practices of peritoneal dialysis clinicians

The aim of this survey is to explore global peritoneal dialysis exercise and physical activity to inform peritoneal dialysis exercise recommendations. By completing this survey, you are consenting to your de-identified information being analyzed. If you wish to receive a copy of the results, please insert your email address at the end of this survey. This survey has been approved by the Global Renal Exercise Network Organizing Committee and the XXXX Human Research Ethics Committee

1. Demographics: Country, Gender, Occupation, Years working in peritoneal dialysis, peritoneal dialysis vintage
2. Who advises patients on exercise/physical activity? Please check all that apply.
3. Is an exercise professional (physiotherapist, physical therapist, kinesiologist, exercise physiologist) involved in the care of the peritoneal dialysis patients in your primary program?
4. In your program do you provide recommendations for: Lifting/Swimming or Water Sports/Activity following catheter insertion/Falls prevention
5. It is important for peritoneal dialysis patients to be physically active (Strongly Agree/Agree/Neutral/Disagree/Strongly Agree)
6. I promote participation in exercise or physical activity to peritoneal dialysis patients (SA/A/N/D/SA)
7. I am confident in prescribing exercise for my peritoneal dialysis patients (SA/A/N/D/SA)
8. Peritoneal dialysis patients can benefit from a structured exercise program (SA/A/N/D/SA)
9. Abdominal strengthening exercises can be safely performed by peritoneal dialysis patients (SA/A/N/D/SA)
10. The majority of peritoneal dialysis patients are physically active (SA/A/N/D/SA)
11. The majority of peritoneal dialysis patients could perform more exercise (SA/A/N/D/SA)
12. The majority of patients would benefit from an exercise professional in my peritoneal dialysis program (SA/A/N/D/SA)
13. What are the three (3) most important areas you would like to see covered in an exercise program for peritoneal dialysis patients?

If you wish to make any comments related to exercise and physical activity in peritoneal dialysis please comment below

Supplementary Table 2. EQUATOR Consensus-Based Checklist for Reporting of Survey Studies (CROSS)

Section/topic	Item	Item description	Reported on page #
**Title and abstract**			
Title and abstract	1a	State the word "survey" along with a commonly used term in title or abstract to introduce the study's design.	1,3
	1b	Provide an informative summary in the abstract, covering background, objectives, methods, findings/results, interpretation/discussion, and conclusions.	3
**Introduction**			
Background	2	Provide a background about the rationale of study, what has been previously done, and why this survey is needed.	4
Purpose/aim	3	Identify specific purposes, aims, goals, or objectives of the study.	4
**Methods**			
Study design	4	Specify the study design in the methods section with a commonly used term (e.g., cross-sectional or longitudinal).	5
Data collection methods	5a	Describe the questionnaire (e.g., number of sections, number of questions, number and names of instruments used).	5,6
	5b	Describe all questionnaire instruments that were used in the survey to measure particular concepts. Report target population, reported validity and reliability information, scoring/classification procedure, and reference links (if any).	5
	5c	Provide information on pretesting of the questionnaire, if performed (in the article or in an online supplement). Report the method of pretesting, number of times questionnaire was pre-tested, number and demographics of participants used for pretesting, and the level of similarity of demographics between pre-testing participants and sample population.	5
	5d	Questionnaire if possible, should be fully provided (in the article, or as appendices or as an online supplement).	Online Suppleme nt
Sample characteristics	6a	Describe the study population (i.e., background, locations, eligibility criteria for participant inclusion in survey, exclusion criteria).	5
	6b	Describe the sampling techniques used (e.g., single stage or multistage sampling, simple random sampling, stratified sampling, cluster sampling, convenience sampling). Specify the locations of sample participants whenever clustered sampling was applied.	5,6
	6c	Provide information on sample size, along with details of sample size calculation.	6. SSC N/A
	6d	Describe how representative the sample is of the study population (or target population if possible), particularly for population-based surveys.	5
Survey administration	7a	Provide information on modes of questionnaire administration, including the type and number of contacts, the location where the survey was conducted (e.g., outpatient room or by use of online tools, such as SurveyMonkey).	5
	7b	Provide information of survey's time frame, such as periods of recruitment, exposure, and follow-up days.	5,6

	7c	Provide information on the entry process:<br>–>For non-web-based surveys, provide approaches to minimize human error in data entry.<br>–>For web-based surveys, provide approaches to prevent "multiple participation" of participants.	N/A<br><br>6
Study preparation	8	Describe any preparation process before conducting the survey (e.g., interviewers' training process, advertising the survey).	N/A
Ethical considerations	9a	Provide information on ethical approval for the survey if obtained, including informed consent, institutional review board [IRB] approval, Helsinki declaration, and good clinical practice [GCP] declaration (as appropriate).	5
	9b	Provide information about survey anonymity and confidentiality and describe what mechanisms were used to protect unauthorized access.	5
Statistical analysis	10a	Describe statistical methods and analytical approach. Report the statistical software that was used for data analysis.	6
	10b	Report any modification of variables used in the analysis, along with reference (if available).	N/A
	10c	Report details about how missing data was handled. Include rate of missing items, missing data mechanism (i.e., missing completely at random [MCAR], missing at random [MAR] or missing not at random [MNAR]) and methods used to deal with missing data (e.g., multiple imputation).	6
	10d	State how non-response error was addressed.	6
	10e	For longitudinal surveys, state how loss to follow-up was addressed.	N/A
	10f	Indicate whether any methods such as weighting of items or propensity scores have been used to adjust for non-representativeness of the sample.	11
	10g	Describe any sensitivity analysis conducted.	N/A
**Results**			
Respondent characteristics	11a	Report numbers of individuals at each stage of the study. Consider using a flow diagram, if possible.	N/A
	11b	Provide reasons for non-participation at each stage, if possible.	N/A
	11c	Report response rate, present the definition of response rate or the formula used to calculate response rate.	6
	11d	Provide information to define how unique visitors are determined. Report number of unique visitors along with relevant proportions (e.g., view proportion, participation proportion, completion proportion).	N/A
Descriptive results	12	Provide characteristics of study participants, as well as information on potential confounders and assessed outcomes.	Table 2
Main findings	13a	Give unadjusted estimates and, if applicable, confounder-adjusted estimates along with 95% confidence intervals and p-values.	Tables 3 and 4
	13b	For multivariable analysis, provide information on the model building process, model fit statistics, and model assumptions (as appropriate).	6
	13c	Provide details about any sensitivity analysis performed. If there are considerable amount of missing data, report sensitivity analyses comparing the results of complete	N/A

		cases with that of the imputed dataset (if possible).	
**Discussion**			
Limitations	14	Discuss the limitations of the study, considering sources of potential biases and imprecisions, such as non-representativeness of sample, study design, important uncontrolled confounders.	10, 11
Interpretations	15	Give a cautious overall interpretation of results, based on potential biases and imprecisions and suggest areas for future research.	9, 10
Generalizability	16	Discuss the external validity of the results.	11
**Other sections**			
Role of funding source	17	State whether any funding organization has had any roles in the survey's design, implementation, and analysis.	12
Conflict of interest	18	Declare any potential conflict of interest.	12
Acknowledgements	19	Provide names of organizations/persons that are acknowledged along with their contribution to the research.	12

Supplementary Table 3. Distribution of exercise perception outcomes by covariates-Part 1 (N=1125)

	It is important for PD patients to be physically active			I promote participation in exercise or physical activity to PD patients			I am confident in prescribing exercise for my PD patients			PD patients can benefit from a structured exercise program		
	SDA/DA	SA/A	Neutral	SDA/DA	SA/A	Neutral	SDA/DA	SA/A	Neutral	SDA/DA	SA/A	Neutral
Gender												
Male	0	310	5	6	266	43	49	186	80	0	294	21
Female	5	796	9	12	741	57	130	496	184	6	755	49
Occupation												
Dietitian	0	59	0	2	52	5	23	15	21	1	53	5
EP	0	28	0	0	25	3	1	25	2	0	28	0
Nephrologist	2	441	5	6	400	42	73	268	107	2	417	29
Nurse	3	546	9	10	501	47	78	358	122	3	519	36
Social worker	0	14	0	0	14	0	1	5	8	0	14	0
Other	0	18	0	0	15	3	3	11	4	0	18	0
Work experience												
<2 years	1	99	3	4	87	12	19	49	35	2	92	9
2-5 years	0	199	4	4	181	18	36	115	52	0	192	11
>5 years	1	808	7	10	739	70	124	518	177	4	765	50
Centre size												
<20 patients	1	192	2	5	163	27	41	101	53	3	178	14
20-50 patients	0	311	3	4	286	24	50	198	66	1	283	30
51-100 patients	1	254	3	5	227	26	45	152	61	0	246	12
>100 patients	3	349	6	4	331	23	43	231	84	2	342	14
Income status												
High income	1	676	5	15	592	75	132	351	199	2	623	57
Upper middle	4	349	9	3	337	22	36	277	49	4	347	11
Lower middle	0	81	0	0	78	3	11	54	16	0	79	2

EP = Exercise professional; SDA=Strongly disagree; DA=Disagree; A=Agree; SA=Strongly agree

Supplementary Table 3. Distribution of exercise perception outcomes by covariates-Part 2 (N=1125)

	Abdominal strengthening exercises can be safely performed by peritoneal dialysis patients			The majority of peritoneal dialysis patients are physically active			The majority of peritoneal dialysis patients could perform more exercise			The majority of patients would benefit from an exercise professional in my peritoneal dialysis program		
	SDA/DA	SA/A	Neutral	SDA/DA	SA/A	Neutral	SDA/DA	SA/A	Neutral	SDA/DA	SA/A	Neutral
Gender												
Male	15	203	97	125	113	77	11	259	45	6	258	51
Female	106	448	256	323	304	183	57	646	107	31	690	89
Occupation												
Dietitian	4	21	34	46	5	8	5	48	6	2	53	4
EP	0	21	7	14	8	6	0	26	2	0	25	3
Nephrologist	26	299	123	172	177	99	18	368	62	8	380	60
Nurse	89	295	174	201	216	141	44	436	78	27	461	70
Social worker	1	6	7	6	5	3	0	13	1	0	13	1
Other	1	9	8	9	6	3	1	14	3	0	16	2
Work experience												
<2 years	11	54	38	32	36	35	3	83	17	2	88	13
2-5 years	26	119	58	101	69	33	12	160	31	4	178	21
>5 years	84	478	257	315	312	192	53	662	104	31	682	106
Centre size												
<20 patients	20	109	66	71	79	45	14	142	39	6	168	21
20-50 patients	34	174	106	135	95	84	15	259	40	12	263	39
51-100 patients	23	154	81	112	95	51	12	216	30	4	222	32
>100 patients	44	214	100	130	148	80	27	288	43	15	295	48
Income status												
High income	61	357	264	340	156	186	31	568	83	19	575	88
Upper middle	51	247	64	88	211	63	30	281	51	17	299	46
Lower middle	9	47	25	20	50	11	7	56	18	1	74	6

EP = Exercise professional; SDA=Strongly disagree; DA=Disagree; A=Agree; SA=Strongly agree
